# Human embryonic stem cell cultivation: historical perspective and evolution of xeno-free culture systems

**DOI:** 10.1186/s12958-015-0005-4

**Published:** 2015-02-22

**Authors:** Nina Desai, Pooja Rambhia, Arsela Gishto

**Affiliations:** Department of Obstetrics and Gynecology, Cleveland Clinic, Beachwood, OH USA

**Keywords:** Embryonic stem cells, Human, Feeder layers, 3-D culture, Hydrogel, Cell adhesion, ECM (extracellular matrix), Tissue regeneration, Differentiation, Pluripotent

## Abstract

Human embryonic stem cells (hESC) have emerged as attractive candidates for cell-based therapies that are capable of restoring lost cell and tissue function. These unique cells are able to self-renew indefinitely and have the capacity to differentiate in to all three germ layers (ectoderm, endoderm and mesoderm). Harnessing the power of these pluripotent stem cells could potentially offer new therapeutic treatment options for a variety of medical conditions. Since the initial derivation of hESC lines in 1998, tremendous headway has been made in better understanding stem cell biology and culture requirements for maintenance of pluripotency. The approval of the first clinical trials of hESC cells for treatment of spinal cord injury and macular degeneration in 2010 marked the beginning of a new era in regenerative medicine. Yet it was clearly recognized that the clinical utility of hESC transplantation was still limited by several challenges. One of the most immediate issues has been the exposure of stem cells to animal pathogens, during hESC derivation and during in vitro propagation. Initial culture protocols used co-culture with inactivated mouse fibroblast feeder (MEF) or human feeder layers with fetal bovine serum or alternatively serum replacement proteins to support stem cell proliferation. Most hESC lines currently in use have been exposed to animal products, thus carrying the risk of xeno-transmitted infections and immune reaction. This mini review provides a historic perspective on human embryonic stem cell culture and the evolution of new culture models. We highlight the challenges and advances being made towards the development of xeno-free culture systems suitable for therapeutic applications.

## Background

Human embryonic stem cells (hESCs) have emerged as exciting candidates for cell therapies in regenerative medicine due to their capacity to self-renew and differentiate into lineages of all three embryonic germ layers. Studies have demonstrated the ability of hESCs to differentiate into a number of pathologically relevant cell types, including insulin-producing cells [1], neural precursor cells [2], cardiomyocytes [3], and hepatocyte-like cells [4], highlighting their potential to be used as a renewable cell source to treat major diseases such as type I diabetes, Parkinson’s disease, cardiovascular disease and liver diseases, among many others. Significant research efforts have thus been put towards human pluripotent stem cell research for advancements in drug development, cell regeneration and delivery of gene therapies. To reach the full therapeutic potential of hESCs, defined and reproducible culture systems must be integrated in order to generate quantities of hESCs and their derivatives that are able to sustain therapeutic applications.

Early studies with human ESCs involved direct culture on mouse embryonic fibroblast feeder cells (MEFs) or animal derived extracellular matrices with conditioned medium from MEF feeder cells in medium supplemented with fetal bovine serum (FBS) [5,6]. This exposure to non-human (xeno) cells and biologics left hESCs vulnerable to xeno-contamination and immune rejection, ultimately rendering many of the existing hESC lines unsuitable for clinical transplantation. This mini review provides a historical perspective on human embryonic stem cell culture and the evolution of xeno-free culture systems that will ultimately advance the development of clinical grade hESC lines suitable for therapeutic applications.

## Review

Human pluripotent cells (hPSCs) include not only embryonic stem cells (hESC) but also human induced pluripotent cells (hiPSCs). The latter are derived from adult somatic cells that have been reprogrammed genetically to behave like embryonic stem cells, expressing genes necessary to maintain pluripotency. They represent an additional valuable source of stem cells for therapeutic use as they have the potential to differentiate in to any cell lineage. Although this mini-review concentrates on embryo-derived stem cells many of the described culture systems have also been applied to hiPSCs.

### Properties of human embryonic stem cells

The functional definition of embryonic stem cells includes four criteria: 1) Origin from a pluripotent cell population 2) Capable of self-renewal indefinitely in the undifferentiated state 3) Capable of maintaining normal karyotype during growth 4) Clonally derived cells capable of differentiation in to all three embryonic germ layers in vitro or in to teratomas in vivo. In culture, hESC appear as tightly packed colonies with distinct borders. Within the colonies, individual stem cells have a high nucleus: cytoplasm ratio with distinctive nucleoli. Human stem cell identification and characterization in vitro also includes demonstration of a high level of alkaline phosphatase activity and expression of specific embryonic stem cell markers [7]. The pluripotent hESCs express transcription factors Oct 4, Nanog and Sox 2 as well as tumor rejection antigens Tra–1–81 and Tra–1–60. They are also strongly positive for stage-specific-embryonic antigens SSEA 3 and SSEA 4 but negative for SSEA 1. It is important that any newly derived hESC line be rigorously tested to unequivocally identify it as pluripotent. This should also include demonstration of differentiation capability and positive identification of all three germ layers in embryoid bodies formed in vitro or by teratoma formation in vivo. Karyotypic stability during culture needs also to be established.

### Derivation of human embryonic stem cell lines

Human embryonic stem cells are typically derived from the inner cell mass (ICM) of blastocyst stage embryos. These unique cells can continuously proliferate and also differentiate in to all three embryonic germ layers. The establishment of the first hESC lines [5] provided the impetus for the derivation of over a thousand of other hESC lines, worldwide [8]. Human embryonic stem cell lines have been derived from the inner cell mass (ICM) of fresh as well as frozen blastocyst stage embryos. The pluripotent ICM can be isolated via mechanical dissection [9,10], laser dissection [11,12] or immunosurgery [5,13,14]. In the latter technique, polyclonal anti-human serum combined with complement mediated lysis using guinea pig serum is used to selectively lyse trophectodermal cells, leaving behind only the ICM. This immunosurgery technique is perhaps the least suitable for generation of clinical grade hESC lines since it involves the use of non-human antibodies and proteins. Strom et al. successfully derived hESC lines from ICM isolated by micro dissection of human blastocysts using fine needles. Laser-assisted biopsy of the blastocyst is however emerging as the most promising technique for xeno-free isolation of the ICM [12,15]. Subsequent to ICM isolation, the pluripotent stems cells are plated and cultivated to generate the ESC line. Methodologies for propagation of these isolated pluripotent stem cells have included feeder layers, extracellular matrices, proteins/peptides as well as synthetic polymers.

The destruction of embryos for ICM isolation has however raised ethical concerns [16]. An innovative alternate approach has been generating hESC lines from single blastomeres biopsied from patient embryos as is routinely done during preimplantation genetic testing (PGD) [17-19]. Chung et al. successfully derived 5 human ESC lines from a single blastomere biopsied from individual eight cell embryos [17]. The single blastomere was co-cultured with the parental biopsied embryo for up to 24 hours before moving to blastocyst medium containing fibronectin and laminin. The biopsied parental embryo remained available for clinical use after cultivation to the blastocyst stage. The presence of laminin was noted to be critical for formation of ESC-like aggregates and prevention of trophectoderm-like vesicles. During stem cell derivation in serum-free media, inclusion of FGF increased cloning efficiency, sustained cell proliferation and was necessary to prevent differentiation of hESC [20,21].

Efficiency of human ESC line generation from individual blastomeres has been variable dependent not only on culture conditions to retain “plasticity” or totipotency of the isolated blastomere but also on properties of the isolated blastomere and the embryonic cell stage of isolation. Early efforts to generate ESC lines from morula stage embryos were unfruitful mostly resulting in aggregates of trophoblast-like cells. Blastomere isolation from embryos at earlier cell stages (4 and 8-cells) before cell polarization into outer TE cells and inner non-polarized cells destined to become the ICM were more successful. Initial reports suggested that hESC lines derived from different developmental stages possessed distinct gene expression profiles. More recent data however, demonstrate that hESCs do in fact display a consistent gene profile and preserve a similar pluripotent phenotype, regardless of the source of derivation, ie. ICM-derived hESC and blastomere derived hESC. Slight differences in both the molecular and phenotypic profiles observed between cell lines are most likely attributed to minor differences in derivation and culture protocols [22,23].

### Culture systems for embryonic stem cell propagation

#### Feeder cell monolayers

Co-culture of isolated stem cells with mouse embryonic fibroblasts (MEFs) was the first technique used for successful establishment of hESC lines [5,14]. These initial hESC lines were derived from ICM’s isolated from human blastocysts using immunosurgery. Growth factors and cytokines secreted by MEF cells were found to be necessary to maintain the pluripotent state of the stem cells through repeated passages and time in culture. Human stem cell lines were shown to express the transcription factor Oct-4 associated with pluripotency of the ICM and down regulated upon cell differentiation [14]. These first successful demonstrations of hESC line derivation using mouse feeder layers have served as the basis for understanding stem cell biology and culture requirements for maintenance of pluripotency.

The transmission of animal-derived infectious pathogens associated with MEFs remains however a critical concern that precludes the use of current hESC lines for clinical transplantation purposes. MEF cells can contain viral particles capable of infecting humans [24]. Also problematic is the use of bovine serum in these systems, potentially exposing the cell line to prion transmission as well as bovine viruses [25]. Transmission of xeno-derived molecules can also occur through membrane derived micro vesicles that typically function in cell: cell communication [26,27]. Any pathogenic molecules taken up in to the microvesicles would be capable of contaminating the entire hESC culture. This problem may persist even if cells are later transitioned to a feeder-free and animal-protein free culture system. The self-renewing capacity of stem cells demands stringent controls during therapeutic use since cells from a single hESC line could potentially be transplanted into many patients, increasing the risk of inadvertently transmitting infectious pathogens to a large patient population. Bioactive molecules such as non-human sialic acid Neu5GC found in mouse feeders as well as animal-derived serum products can also present a problem. This animal derived sialic acid can be metabolically incorporated on to the cell surface of hESCs [28]. Since humans have circulating antibodies to Neu5GC, this raises the risk of an immune response after hESC transplantation.

Human feeder layers for hESC culture were therefore explored as a first step towards the ultimate goal of xeno-free culture models. To this end a variety of different human cell types such as human fallopian tube cells [29], fetal foreskin [30], fetal muscle and skin [31], transgenic fetal liver stromal cells [32], bone marrow [33], umbilical cord [34], placental cells [35,36] and endometrial cells [37-39] have been tested for ability to support stem cell renewal. From these studies it was clear that both fetal and adult cells could be successively used. The human umbilical stromal cell culture model offers a large source of cells that can be collected noninvasively and side-steps some of the ethical concerns raised with the use of fetal foreskin, muscle or bone marrow derived feeder layers. The use of established cell lines as feeders rather than primary cultures may be less labor-intensive and allows more consistency in feeder layer preparation. New hESC lines have been derived and propagated using a commercially available human foreskin fibroblast (HFF) line [40,41]. Undifferentiated hESC growth in xeno-free culture media for over 80 passages was possible with this HFF feeder [41,42]. Endometrial cells with their unique role in supporting ICM proliferation and pluripotency in vivo, also proved to be effective for in vitro culture of stem cells [38,39,43]. Autogenic feeder layers derived from the human stem cell line being cultured was another interesting approach [44,45]. Such culture systems entirely eliminated the risk of allogeneic pathogen contamination from a donor feeder layer. Chen et al. [44] generated fibroblast-like cells from the commercially available H9 hESC line and went on to use these cultures as a feeder layer for cultivation of the H9 stem cells. Indirect co-culture by separating hESC from the feeder cell population with a microporous membrane also proved successful and reduced the risk of cross-contamination [43,46].

These early studies with feeder layers have contributed much to our understanding of the behavior, characteristics and conditions necessary for stem cell renewal in vitro. Basic fibroblast growth factor (bFGF) was determined to be a key growth factor for maintaining hESC pluripotency and shown to be endogenously produced by human feeder cells used for hESC culture [32,47,48]. During stem cell derivation in the absence of serum, inclusion of bFGF increased cloning efficiency from single blastomeres, sustained cell proliferation and was necessary to prevent differentiation of hESC [20,21]. bFGF supports hESC renewal by promoting expression of IGF-II, TGFβ and Activin A (a member of the TGFβ superfamily) [49]. TGFβ and Activin A signaling modulate cell fate decisions in the early embryo and are involved in maintaining the pluripotency of inner cell mass cells [49-54]. Conditioned medium from feeder cells contain high levels of Activin A [51]. Hongisto et al. showed that feeder cell expression of a specific isoform of laminin (LM-511) was associated with its functionality in supporting hESC [55].

Despite the aforementioned successes, feeder cell dependent hESC culture systems have many limitations that make them unsuitable for future therapeutic applications. Maintenance of feeder layers is very labor intensive with lot-to-lot inconsistencies between feeder populations. This variation can ultimately affect the stem cells as well as their downstream differentiation in to specific cell types for clinical therapy.

#### Feeder-free culture systems

The focus of current stem cell research has been the design of feeder-free, xeno-free culture systems with chemically defined media formulations. We present some of the most relevant studies in the ensuing sections. Tables 1 and 2 summarize the more recent work on hESC culture and contrast the different culture systems. Systems classified as “xeno-free” will likely prove the most attractive for future clinical therapeutic applications.

**Table 1:** **Feeder-free culture systems using extracellular matrix (ECM) proteins or biological substrates**

**System description/substrate**	**Cell type**	**Medium/supplements**	**SF** ^**1**^	**CD** ^**2**^	**XF** ^**3**^	**Culture period**	**Ref.**
*Matrigel-mouse*	hESC	MEF –CM^4^ (basal medium DMEM+ KSR^5^) bFGF	Y	N	N	180 days	[6]
*Matrigel-mouse*	hESC/	DMEM/F12	Y	N	N	20 passages	[102]
	hiPSC	Dorsomorphin, IWP-2, bFGF,, TGF-β1, Activin					
*ECM from embryoid body (e-ECM)*	hESC	TeSR2	Y	Y	Y	20 passages	[65]
*Human fibronectin*	hESC	DMEM/KSR	Y	N	N	>47 passages	[69]
		bFGF,, TGF-β1 (+/− LIF)				>180 days	
*Laminin*	hESC	DMEM/KSR	Y	N	N	>20 passages	[51]
		bFGF, Activin					
*Laminin,vitronectin, fibronctin and collagen IV (defined human ECM)*	hESc	TeSR1	Y	N	Y	11–25 passages	[70]
*Human placental ECM*	hESC	XF medium -Human plasma extract, with DMEM/F12 base, bFGF, TGFβ	N	N	Y	39 passages	[68]
*Laminin isoforms 111,332,511*	hESC/	MEF –CM (basal medium DMEM+ KSR^5^)	N	N	N	10 passages	[72]
	hiPSC						
		bFGF					
*Laminin E8 fragments (LM-E8)*	hESC/	mTeSR1,	Y	N	N		[74]
	hiPSC	StemPro,	Y	N	N	10–30 passages	
		TeSR2	Y	Y	Y		
*E-cadherin*	hESC	mTeSR1	Y	N	N	>90 days	[79]
	hiPSC						
*Laminin-511*	hESC	TeSR1	Y	N	Y	20 passages	[71]
*Laminin-521/E-cadherin matrix*	hESC	mTeSR1,	Y	N	N	35 passages	[80]
	hiPSC	TeSR2,	Y	Y	Y	12–15 passages	
*Vitronectin*	hESC	mTeSR1	Y	N	N	10 passages	[82]
*Vitronectin- truncated*	hESC	E8 medium	Y	Y	Y	>25 passages	[83]
	hiPSC						
*Fibronectin,, laminin and vitronectin*	hESC	DMEM/F12 +/− bFGF	Y	Y	Y	>24 passages	[87]
		Wnt/ID8					
*Recombinant human vitronectin*	hESC/	E8 medium	Y	Y	Y	>30 passages	[85]
*Alginate microencapsulation*	hESC	MEF-CM (basal medium DMEM//KSR)	Y	N	N	14 days	[126]
*Alginate encapsulation of hESC on*		bFGF	Y	N	N	19 days	
*Matrigel coated microcarriers*							
*Aliginate-chitosan scaffold-3D*	hESC	DMEM/F12 + KSR + FBS + bFGF	Y	N	N	21 days	[114]
*Alginate-chitosan microfiber scaffold-3D*	hiPSC	mTeSR1	Y	N	N	10 passages	[115]
		ROCK inhibitor					
*Alginate encapsulated*	hESC	DMEM + KSR + bFGF	Y	N	N	>260 days	[116]
*Nanofibrillar cellulose*	hESC	mTeSR1	Y	N	N	>26 days	[117]
*Aggregates/ in suspension Fibronectin and laminin present*	hESC	Neural basal medium.Neutrodoma-CS	Y	N	Y	>10 weeks	[128]
		bFGF, Activin, Neurotrophins BDNF, NT3, NT4					
*Aggregates in suspension*	hESC	mTESR1	Y	N	N	20–50 passages	[131]
*Polymer to prevent fusion/*	hiPSC/	StemPro	Y	N	N		
*DE-53 Matrigel coated cellulose microcarrier*	hESC	mTeSR1	Y	N	N	25 passages	[125]
		StemPro	Y	N	N		
*Artificial spider silk + vitronectin*	hESC/	Nutirstem XF/FF	Y	N	Y	>30 passages	[93]
	hiPSC						

^1^SF serum –free medium.

^2^CD chemically defined medium (excludes any media containing BSA or HSA fraction due to their unknown and variable composition).

^3^XF xeno-free, no animal components in medium or culture system.

^4^MEF-CM mouse embryonic fibroblast conditioned medium.

^5^KSR knock out serum replacement protein.

**Table 2:** **Synthetic substrates for hESC culture**

**System description/substrate**	**Cell type**	**Medium/supplements**	**SF** ^**1**^	**CD** ^**2**^	**XF** ^**3**^	**Culture period**	**Ref**
*Synthetic acrylate surfaces (PAS) with peptides: Vitronectin, bone sialo protein*	hESC	X -VIVO 10	Y	N	Y	>10 passages	[89]
		bFGF,TGF-β1					
		Activin A					
*Synthemax* ^*4*^	hiPSC	mTeSR1	Y	N	N	10 passages	[90]
*Synthemax* ^*4*^	hESC	mTESR1	Y	N	N	10–20 passages	[91]
	hiPSC	TESR2	Y	Y	Y		
		PSGro	Y	Y	Y		
		Nutristem XF	Y	N	Y		
*PMEDSAH* ^*5*^ *coated plates*	hESC	MEF-CM^6^	N	N	N	25 passages	[98]
		hCCM^7^	N	N	Y		
		StemPro,	Y	N	N		
		mTeSR	Y	N	N		
*Alkanethiol with heparin binding proteins*	hESC	mTeSR1	Y	N	N	>17 passages	[92]
		Rock inhibitor					
*PMVE-alt-MA* ^*8*^	hiPSC	StemPro	Y	N	N	5 passages	[94]
*APMAAm* ^*9*^ *hydrogels*	hESC	mTeSR1	Y	N	N	20 passages	[95]
*Polyethylene glycol hydrogels (PEG)*	hESC	DMEM/F12 + KSR	N	N	N	9 days	[118]
		bFGF					
*Hillex* ^*10*^ *microcarrier//suspension culture*	hESC	KSR-XF 10	Y	N	Y	6–14 days	[123]
		BRASTEM,	Y	N	Y		
		bFGF					

^1^SF serum –free medium.

^2^CD chemically defined medium (excludes any media containing BSA or HSA fraction due to their unknown and variable composition).

^3^XF xeno-free, no animal components in medium or culture system.

^4^Synthemax acrylate surface coated with peptide derived from vitronectin.

^5^PMEDSAH Poly [2-(methacryloyloxy) ethyl dimethyl-(3-sulfopropyl) ammonium hydroxide]—.

^6^MEF-CM mouse embryonic fibroblast conditioned medium.

^7^Human cell conditioned medium.

^8^PMVE-alt-MA poly (methyl vinyl ether-alt-maleic anhydride.

^9^APMAAm aminopropylmethacrylamide—.

^10^Hillex polystyrene beads modified with cationic trimethyl ammonium.

##### Extracellular matrix and cell adhesion molecules

One of the first approaches tested for feeder-free growth relied on the use of extracellular matrix proteins in combination with growth factor supplementation to create an in vitro microenvironment that promoted stem cell renewal. The extracellular matrix, Matrigel isolated from mouse Engelbreth-Holm-Swarm teratocarcinoma cells was used by Xu et al. [6] as a feeder cell substitute for hESC culture. Matrigel is a gelatinous mixture of collagen IV, laminin, proteoglycans, entactin and other growth factors [56]. To date Matrigel, often in combination with growth factors or conditioned medium from feeder cultures has been the most frequently used system for hESC culture [57-63]. Lot-to-lot variations in the Matrigel composition can however pose problems, inhibiting its effectiveness in maintaining cells in a pluripotent state, thus affecting reproducibility of subsequent stem cell differentiation protocols. The use of Matrigel also raises clinical concerns as certain batches have been found to be contaminated with the single stranded mouse RNA virus, Lactate Dehydrogenase Elevating Virus (LDEV), ultimately questioning the safety of using stem cells that have been cultured on animal derived extracellular matrix in a therapeutic setting [64].

Fu et al. extracted the extracellular matrix from embryoid bodies (e-ECM) derived from the H9 ESC line [45]. This human-derived ECM consisting of fibronectin, laminin and collagen type IV, proved to be a viable method for xeno-free propagation of hESCs used to generate a xeno-free autologous feeder-free culture system for long term culture of the H9 hESC line. The hESCs were successfully maintained for 20 passages on this ECM substrate in TeSR2, a chemically defined serum-free and animal protein free medium [65,66]. Spontaneous differentiation in hESCs cultured on e-ECM was markedly less than on Matrigel, suggesting a more supportive environment. This xeno-free culture model was also successfully used for terminal differentiation of the hESCs in to keratinocytes for potential therapeutic application [67]. Human placenta–derived ECM has also been successfully used for hESC culture [68]. Cultures showed genetic stability after 40 passages and differentiation potential of the stem cells was retained.

These studies with extracellular matrix have been instrumental in identifying biomolecules involved in stem cell renewal. Inconsistencies in ECM preparations even if of human origin, do however still present a problem. Certainly, use of chemically defined matrices and recombinant proteins based on ECM may be a more reliable approach towards xeno-free culture of therapeutic grade hESC lines. Xu and colleagues analyzed the individual components of Matrigel and determined that not all are equally effective for sustaining undifferentiated growth of ESC lines [6]. Laminin appeared to be superior to fibronectin and collagen IV for feeder-free growth of stem cells in MEF-conditioned medium [6]. Others have successfully used human fibronectin with bFGF and TGFβ to culture hESC for over 180 days [69]. Beattie et al. [51] showed that medium supplementation with Activin could be used to eliminate the need for MEF cell conditioned medium. Successful propagation of hESCs in a serum and xeno-free medium, TeSR1 using a combination of human matrix components collagen IV, laminin, vitronectin and fibronectin as a substratum marked a significant milestone in the development of clinically compliant hESC culture models [66,70]. This culture system allowed derivation of a karyotypically normal hESC line (WA-15) and also the undifferentiated propagation of H1 and H9 stem cells under defined conditions without animal products. Inclusion of Rho kinase inhibitor (ROCK) in the serum free medium helped prevent apoptosis in individual cells at passaging.

Laminin with its role in cell adhesion has been extensively studied. Rodin et al. described a xeno-free system for hESC and hiPSC cultivation using a recombinant form of human laminin-511 with a defined medium, TeSR1 [71]. This serum-free culture system allowed hESC cell renewal with genetic stability for 4 months (20 passages). Miyazaki tested different recombinant human laminin isoforms for their ability to serve as scaffolds for hESC cultivation [72]. Laminin isoform binding to integrin receptors α6β1 on hESC appeared to be integral to triggering signaling pathways necessary for continuous self-renewal. Recombinant human laminin isoforms LM- 111, 511 and 332 supported hESC adhesion and undifferentiated cell growth. Further studies showed that even fragments of different recombinant laminin isoforms (LM E8’s) if they contained integrin binding capacity could be used for hESC culture [73]. LM 511-E8 was in fact found to be superior to Matrigel or whole laminin isoforms in promoting cell adhesion and proliferation of stem cells. Stem cells could be cultivated under xeno-free conditions in a chemically defined medium (TeSR2) on the LM511-E8 coated substrate for over 30 passages and retained pluripotency as well as a karyotypic stability. The smaller size of LM-8 s as compared to whole laminin simplifies manufacturing the recombinant product making it more cost-effective. Nakagawa and colleagues reported better attachment of hESC and hiPSC dissociated in to single cells using StemFit media with laminin 511-E8 matrix [74].

E-cadherin, another cell adhesion molecule that binds to the integrin α6β1 also plays a role in cell:cell binding [75]. Poor survival and hESC death after dissociation into individual cells is due to a disruption of E-cadherin signaling [76-78]. Nagaoka et al. cultured hESC and hiPSC cells on plates coated with E-cadherin fused with the IgG FC domain [79]. This feeder-free system with a defined medium, TeSR1 was effective as a substitute for Matrigel. The matrix promoted adhesion and both types of pluripotent stem cells (hiPSC and hESC) retained their stem cell attributes for over 90 days in culture. Supplementation of culture media with E-cadherin in combination with recombinant laminin isoform 521 increased efficiency of hESC line derivation from individual blastomeres as well as the ICMs of blastocysts in chemically defined media under xeno-free conditions [80]. This vital work provides a clinically compliant system for the derivation of new stem lines for regenerative medicine. The E-cadherin/laminin 521 based culture system also allowed robust proliferation of hiPSC cells under xeno-free conditions. A cocktail of specific kinase inhibitors along with bFGF may also be beneficial for propagating single stems cells under feeder-free chemically defined conditions [81].

Amongst ECM components, vitronectin is another protein that has emerged as a promising substratum for long term culture of hESC cell serum-free conditions in defined media [82-85]. Recombinant vitronectin was able to effectively replace Matrigel as a substrate for hESC culture in a defined medium [82]. Chen et al. devised a simplified culture system for hPSC using a truncated recombinant vitronectin coating and a chemically defined, xeno-free and albumin-free medium known as Essential 8 (E-8) [83]. This represents a significant advancement in the movement towards clinically compliant culture models for stem cell applications. Hasegawa et al. [84] identified two small chemical molecules that could replace bFGF in culture media and still sustain human stem cell growth [84]. These molecules are modulators of the Wnt/β-catenin signaling pathway [86-88]. Based on molecular clues present in the environment, this pathway can drive hESC cells towards continued cell renewal as well as differentiation. The combination of Wnt modulators with ECM-based substrata (vitronectin, laminin or fibronectin) allows culture in a chemically defined media without exogenous supplementation with bFGF or TGFβ.

Synthetic substrata for stem cell culture have also been investigated as alternatives to complex ECM matrices. Synthetic peptide acrylate surfaces (PAS) constructed by conjugating peptides derived from the biologically active region of extracellular matrix proteins can sustain the self-renewal and pluripotency of hESCs for over 10 passages in a xeno-free defined medium X-Vivo 10 [89]. In this study, Melkoumian et al. tested acrylate surfaces with bound peptides from laminin, vitronectin, fibronectin or bone sialoprotein. PAS with vitronectin or bone-sialoprotein derived peptides showed the best functionality. These cultured stem cells were also capable of directed differentiation in to cardiomyocytes. Corning Synthemax Surface, a synthetic acrylate surface with RGD peptide containing sequence from vitronectin was shown to support the expansion and directed differentiation of human induced pluripotent cells in defined medium [90]. More recently, investigators demonstrated that Synthemax could also be used for the efficient expansion of not only colonies but also single dissociated hPSCs in several serum-free and xeno-free culture media like TESR2, Nutristem XF and PSGro [91].

Klim et al. [92] systematically screened 18 bioactive peptides bound to different substrata to identify peptide surfaces that could sustain hESC growth under defined conditions. Surfaces with the heparin binding pepetide GKKQRFRHRNRKG isolated from vitronectin were shown to facilitate hESC adhesion and proliferation [92]. This peptide was demonstrated to function by interacting with hESC cell- surface glycoasoaminoglycans. Interestingly, surfaces prepared with only peptides containing the core integrin binding motif (arginine-glycine-aspartic acid, RGD) were inadequate for sustained hESC culture.

Wu et al. recently described a novel synthetic material based on spider silk proteins as a suitable substrate for hESC culture [93]. This artificial matrix composed of recombinant miniature spider silk protein, 4RepCT (~3000 base pairs) can be easily manufactured and formed into films, fiber or foam under sterile conditions. Variants of the matrix were created by fusing it with biological peptides derived from laminin or vitronctin. The investigators cultured both hESC and hiPSC lines on 4RepCT-vitronectin films in xeno-free defined medium Nutristem for over 30 passages without loss of stem cell attributes. The cultured hPSC had the capacity to form teratomas in vivo and to undergo directed differentiation into endoderm and also cardiomyocytes in vitro. The properties of the biomatrix allowed for 2-D as well as 3-D culture.

The cost of peptide synthesis, stability of substratum with attached biological molecules and anticipated difficulties with scale up during manufacture has triggered interest in establishing purely synthetic surfaces for serum-free culture of hESC. This has proved to be particularly challenging. Numerous polymer based synthetic surfaces such as PMEDSAH (poly [2- (methyacryloyoxy) ethyldimethyl-(3-sulfopropyl) ammonium hydroxide]), PMVE-alt-MA (poly (methyl vinyl ether-alt-maleic anhydride)), HIT9 and APMAAm (aminopropylmethacrylamide have been tested [89,94-99]. Amongst the tested synthetic polymers, PMEDSAH [98] and APMAAm [95] appear to have the most promise, supporting growth of several hESC lines for at least 20 passages with retention of pluripotency and karyotypic stability.

##### Serum-free culture media, growth factors and other molecular modulators

Transition towards xeno-free culture systems has necessitated the formulation of new media suitable for cultivation of stem cells under serum-free conditions [66,70,83,100,101]. Basal medium such as DMEM/F12 containing amino acids, glucose, vitamins, insulin, transferrin, selenium in a balanced salt solution has been the foundation for many of the in-house as well as commercially available culture media for hESC cultivation. Cholesterol, lipids, GABA, pipecolic, ascorbic acid and β mercaptoethanol are also often included. The compositions of several commercially available media are shown in Table 3. In the absence of serum, all either contain or require bFGF supplementation at concentrations ranging from 20–100 ng/ml. Other select growth factor/cytokine additives used in the various media formulations are TGFß, Activin A, LIF, stem cell factor (SCF) and a structurally similar cytokine FLT3 (FMS-like tyrosine kinase). Under xeno-free conditions, suppression of signaling by bone morphogenetic protein (BMP) can prevent loss of stem cell pluripotency [59,62]. BMP-antagonists like Noggin when included in culture media work synergistically with bFGF to maintain stem cell homeostasis. Another inhibitor of BMP signaling, dorsopmorphin along with inhibitors of the Wnt signaling pathway also work to suppress spontaneous differentiation in pluripotent cells in a xeno-free culture medium [102].

**Table 3:** **Commercially available serum-free media for hESC and hiPSC culture**

**Medium**	**Source (Catalog#)**	**Basal medium/protein/select supplements**	**XF**	**CD**
*ESF*	Cell Science & Technology	ESF basal medium// FAF-BSA conjugated with oleic acid	N	N
		FGF-2, LIF, insulin, transferrin, selenium, ascorbic acid, β-mercaptoethanol		
*mTeSR1*	Stemcell Technologies (#05850)	DMEM/F12/BSA	N	N
		bFGF, TGFβ, insulin, transferrin, cholesterol, lipids, pipecolic acid, GABA, β-mercaptoethanol		
*TeSR1* ^*a*^	Stemcell Technologies	DMEM/F12 /HSA	Y	N
		bFGF, TGFβ, insulin, transferrin, cholesterol, lipids, pipecolic acid, GABA, β-mercaptoethanol		
*TeSR2*	Stemcell Technologies (#05860)	DMEM/F12 // with recombinant HSA	Y	Y
		bFGF, TGFβ, insulin, transferrin, cholesterol, lipids, pipecolic acid, GABA, β-mercaptoethanol		
*E8*	Stemcell Technologies (#05940)	DMEM/F12	Y	Y
		bFGF, TGFβ, insulin, transferrin, selenium, ascorbic acid		
*TESR-E8*	Stemcell Technologies (#05940)	Medium based on E8 formulation	Y	Y
*Nutristem XF/FF*	Stemgent (#010005)	Basal medium/ HSA	Y	N
		bFGF, TGFβ, insulin, transferrin		
*StemPro*	Life Technologies (#A1000701)	DMEM/F12/BSA	N	N
		bFGF, TGFβ, Activin, transferrin, LR3-IGF1, HRG1β		
*X-Vivo 10*	Lonza (#04380Q)	Basal medium/ HSA-pharmaceutical grade purification	Y	Y
		bFGF, hFLT3, transferrin, β-mercaptoethanol		
*Neutrodoma-CS*	Roche (11363743001)	Neural basal medium.Neutrodoma-CS/HSA	Y	N
		bFGF, Activin, Neurotrophins		
*PluriStem*	Millipore (#SCM130)	DMEM//F12, HSA	Y	N
		Activin-A, TGFβ1, b-FGF, lipids, insulin, transferrin, selenium		
		dorsomorphin (DM), IWP-2		
*HyClone HyCellStem*	GE healthcare life sciences (#SR30003.KT)	DMEM//F12; BSA	Y	N
		bFGF, insulin,		
*PSGro*	Stem RD (#SC500M-1)	DMEM//F12 with recombinant HSA	Y	Y
		bFGF, TGFβ1, insulin,transferrrin, selenium, lipids		
*StemFit*	Ajinomoto Co.	Basal medium, HSA	Y	N
		bFGF		

Table 3 contains a list of commercially available media that can be used for serum free culture of HESCs and hiPSCs.

XF xeno-free, no animal components in medium.

CD chemically defined medium (excludes any media containing BSA or HSA fraction due to their unknown and variable composition).

^a^Original formulation similar to mTESR1 but with HSA fraction. Replaced by TESR2 with recombinant form of HSA.

Commercial protein supplements like KnockOut –Serum Replacement (KSR) are often used in culture systems to replace fetal bovine serum but do in fact still contain animal derived serum albumen. Only a few of the media described are completely xeno-free with human serum albumen (HSA) as a protein source in lieu of bovine serum albumen (BSA). However, since HSA is extracted from different pools of donor serum there are batch-to-batch variations. Also HSA manufacturing guidelines only require a purity of ≥96% so most commercial preparations for cell culture contain other undefined plasma proteins and contaminants from the purification process. To technically be classified as a “chemically defined medium”, all constituents of the medium and their concentrations must be known [103,104]. Media formulations with BSA or HSA extracted from plasma, introduce contaminants that may not always be precisely controlled and therefore do not meet this strict definition. Confusion over this terminology is evident from the literature. Numerous media that were developed with bovine and/or human albumen in lieu of serum have early on been referred to as “defined or “chemically defined” (as compared to serum containing media), if all other media components were quantifiable. Recombinant HSA is now often substituted for serum derived HSA in designing new chemically defined media. Table 3 describes commercially available media for serum-free culture of hESCs and information on their composition. Several xeno-free chemically defined media are currently on the market such as TeSR2 and PSGro with recombinant HSA, as well as Essential 8 (E8) and TESR-E8 which do not contain albumen. X-Vivo 10, a defined medium, with recombinant growth factors and highly purified, traceable pharmaceutical grade HSA is also a part of this grouping.

Although considerable progress has been made in the design of chemically defined xeno-free media, cell passage, and fold-expansion may be limited by the quality of culture supplements and their stability in culture. Daily replenishing of media is often recommended when using chemically defined media and this can be both costly and labor intensive.

##### 3D Culture models

Although chemically defined ECM based matrices have helped advance the design of xeno-free hESC culture models, they may still be limited in their ability to physiologically mimic the in vivo stem cell niche. Studies indicate distinct differences in cell signaling and behavior in cells grown in 2-D versus 3-D culture [105-107]. During embryogenesis, inner cell mass cells are embedded within a dynamic 3D stem cell niche, which helps direct both self-renewal and differentiation via the presentation of specific cell signaling molecules, regulation of matrix stiffness, and the establishment of cytokine gradients [108]. While the exact biomolecular composition of this native hESC environment has not been fully defined to date, it is likely that 2D systems will prove inadequate in functionally mimicking the in vivo microenvironment, ultimately restricting their ability to optimally support downstream differentiation applications. Bioengineered 3-D scaffolds offer an opportunity to modulate stem cell behavior in vitro to more closely resemble that occurring in vivo [109-112].

Numerous substrates for 3-D culture have been explored and tested. As a biomaterial, hydrogels are perhaps the most versatile and have been used in a variety of engineering applications. Hydrogels are a network of polymer chains either of natural or synthetic origin that are very absorbent and flexible due to high water content, thus resembling natural matrices. Hyaluronic acid (HA) is a glycosaminoglycan (GAG) and a primary component of in vivo extracellular matrices, making it particularly attractive in designing 3-D scaffolding for stem cells. GAG’s are known participators in receptor signaling complexes, cell-cell adhesion, and cell-matrix interactions. HA-hydrogel architecture and rigidity can be easily manipulated. HA encapsulated hESCs were shown to retain pluripotency for 20 days in culture and the need for continuous stem cell passaging was eliminated [113]. The hydrogel scaffolds also allowed hESC to be driven along specific paths of differentiation. Stem cell release from the hydrogel necessitated enzymatic digestion with hyaluronidase which may however impact stem cell functionality in the long term. It should be noted MEF-conditioned medium was still used in this study.

Others have used a hydrogel scaffold composed of chitosan, a deceatylated cationic polysaccharide and alginate [114]. Investigators were able to show self-renewal and characteristic hESC gene activity for 21 days in the absence of feeder layers or conditioned medium. Direct implantation of hESC-scaffold constructs in to mice resulted in teratoma formation, with immunohistochemical evidence of all three germ layers. Microfiber constructs of chitosan-alginate also support hESC self-renewal under chemically defined conditions [115]. The 3-D microfiber culture model has the added benefit of allowing direct cryopreservation in situ of encapsulated cells. To date there has only been one report of hESC culture for more than 30 days in a 3-D matrix. Using alginate hydrogels, Siti-Ismail and colleagues [116] were able to maintain hESCs in a pluripotent state for 260 days, without enzymatic treatment and repeated passaging of the cells. This was accomplished using a basic stem cell maintenance medium DMEM with 20% knockout serum replacer and bFGF supplementation.

A plant-derived nanofibrillar cellulose (NFC) has similarly been used to create a flexible 3D environment [117]. This hydrogel system utilized maintained stem cells in culture as 3-D spheroids, under xeno-free and feeder-free conditions in a defined medium, TESR. Passaging was accomplished by enzymatically denaturing the matrix and transferring the cells to new NFC platforms every 7–12 days. The hESC could be maintained undifferentiated as 3-D spheroids for 26 days after which teratoma formation was observed. The undifferentiated hESCs were karyotypically normal and expressed mRNA for all pluripotency markers.

Synthetic polymers are especially valuable for 3-D scaffolding, offering the advantage of versatility. Microfabrication techniques can be used to generate scaffolds with specific chemical signals immobilized on the matrix. As discussed earlier, combining matrices with tissue-specific bioactive molecules such as cell-adhesion proteins, growth factors and peptides are particularly promising. Derivatives of polyethylene glycol (PEG) combined with tissue-specific bioactive molecules such as cell-adhesion proteins, growth factors and peptides are particularly promising. The presentation of physiologically relevant signals allows the hESC microenvironment to be tailored towards self-renewal or differentiation as needed. PEG hydrogel constructs cross-linked with matrix metalloproteinase (MMP)-sensitive peptides and coupled with integrin-specific adhesion ligands are able to sustain the pluripotency of mouse and human stem cells [118,119]. The 3-D PEG-based scaffolds supported the expansion, self-renewal and differentiation potential of pluripotent hESC lines H1, H9 and Novo, under feeder-free conditions in culture medium supplemented with bFGF and knock-out serum replacement. Increased mRNA expression of stemness- related genes were detected in hESCs after 3-D culture on PEG scaffolds as compared to 2-D culture on MEF feeders or in Matrigel.

Suspension culture of hESCs on microcarriers (MC) has also been used to create a pseudo 3D microenvironment that can be easily manipulated to direct cells towards undifferentiated proliferation or directed cell differentiation [94,104,120-127]. Microcarriers allow easy scale up in bioreactors to increase total stem cell production. Cell adhesion to microcarriers can be increased by altering their surface. hESC could be cultured on Matrigel coated cellulose beads in serum-free mTeSR and Stem Pro media for 20 passages without loss of stem cell characteristics [125]. Marinho and colleagues [123] developed a serum-free xeno-free culture system for hESC culture using polystyrene microcarrier spheres positively charged with triethyl-ammonium groups (Hillex II) and a defined medium BRASTEM supplemented with bFGF. High growth rate of stem cells and retention of pluripotency was demonstrated after six days in culture. Although promising, serial passaging of pluripotent hESCs on microcarriers over a much longer time interval are necessary to validate this xeno-free culture model for any clinical application. One of the problems with MC based suspension culture has been the tendency of hESC cells to aggregate resulting in multi-bead clusters with low speed agitation in the bioreactors. High levels of agitation resulted in shear stress which was also detrimental to continued propagation hESC cultures.

Suspension culture of hESCs without the use of coatings or microcarriers is also possible [128-131]. Aggregate cell suspension culture of hESCs has been shown to be a particularly favorable culture system for embryoid body formation, eliminating the need for extraneous scaffolds [130]. Steiner and colleagues devised a culture model for generating hESC lines from clusters of inner cell mass cells kept in suspension culture. The initial ICM were extracted from embryos using a laser. They were able to generate 3 new hESC lines under xeno-free conditions in defined medium supplemented with laminin, fibronectin, bFGF, Activin A and neurotrophins BDNF, NT3 and NT4. The new stem cell lines retained pluripotent characteristics even after 10 weeks in culture, were capable of forming all 3 germ layers and could undergo directed differentiation in to neural spheres. Loss of cell viability during passaging can however result in lower expansion rates in suspension culture as compared to that typically observed with adherent cell culture models. Wang et al. developed a scalable xeno-free and clinically compliant culture system for hiPSC suitable for both suspension and adherent culture [85]. This was achieved in the chemically defined medium, E8 that consists of just 8 components. hiPSC maintained pluripotency in culture expressing all stem cell markers and exhibiting karyotype stability after more than 30 passages. The induced pluripotent cells could be directed towards differentiation into hematopoetic cells and showed teratoma formation upon injection in to mice. Demonstrated efficacy combined with its simplicity make this model especially attractive and cost-effective for large scale production of hiPSCs. A 3-D sphere culture system using a methylcellulose polymer to suppress fusion of aggregates was recently described and may help to further optimize the suspension culture model [131]. Investigators tested the system with both mTESR1 and StemPro medium, with similar results as regards retention of pluripotency.

### Summary

Considerations in selection of optimal matrices for stem cell culture should also include ease of stem cell release for passaging, stability over time and compatibility with sterilization techniques. The latter is often overlooked but is especially critical if substrates containing biological components like growth factors and peptides are to be used. Another consideration is the cost of using exclusively xeno-free synthetic media, recombinant growth factors and other additives. Culture design must also allow for large-scale propagation to meet the stem cell dosages needed for patient treatment cycles. The ability to effectively direct terminal differentiation of stem cells under these same xeno-free conditions is also of paramount importance in developing future therapeutic applications.

### Therapeutic application and clinical trials

Currently, the FDA requires all hESC (and hiPSC) lines intended for use in clinical trials to be developed in compliance with current Good Manufacturing Practices (cGMP) and in accordance with the Guidelines for the Clinical Transplantation of Stem Cells [132]. Manufacturing process, reagents, validation of methodology, environmental controls, safety etc. must all be monitored and documented in detail in accordance with cGMP regulations. Terminal differentiation of the hPSCs in to the desired cell type for treatment needs also to be performed under stringent cGMP compliant conditions to avoid possibility of tumor formation on transplant. Detailed characterization of the cells, demonstration of karyotype stability and function in animal models are necessary prior to any therapeutic application. While regulations do not preclude the use of animal derived products when deemed necessary, it is widely believed that xeno-free culture is the only way to limit the potential risks of hESC transplantation rejection. Most of the hESC lines currently in use have in fact been exposed to potentially immunogenic and pathogenic animal components at some point, whether during their isolation, propagation, or both. To date, only a few groups have produced hESC lines derived under these conditions [15,133,134]. Reproducibility and reliability will be key for any type of therapeutic application. Chemically defined media using only recombinant supplements may be the surest way to maintain the level of consistency and safety desirable in the clinical setting.

FDA approval of the first clinical trials of hESCs for treatment of spinal cord injury and macular degeneration in 2009 marked the beginning of a new era in regenerative medicine [135-141]. Geron Corporation a biotechnology firm based in California planned to inject patients with GRNOPC1, a product developed from human embryonic stem cells. The basis for this treatment was a technique for development of oligodendrocytes from hESC by investigators at the University of California [142]. The first patient treated in October 2010, after a spinal cord injury from a car accident received 1.5 million oligodendrocyte precursor cells. The planned enrollment for the trial was 10 patients but the study was unexpectedly discontinued after the treatment of only 3 more patients. High cost and difficulties producing the necessary dosage of stem cells under clinically compliant conditions may have been the cause. No official data were ever published. However preliminary results indicated that while patients did not experience adverse effects, the treatment did not result in any noticeable improvement to spinal injury or neurological condition.

Advance Cell Technology (Marlborough, Massachusetts) has two on-going clinical trials. The first is for Stargardt’s Macular Dystrophy, which results in blindness from degeneration of the retinal epithelium and loss of photoreceptor cells. The second is for age-related dry macular degeneration. Retinal pigment epithelial cells (RPE) derived from hESC were used to restore the degenerative retinal epithelial monolayer. Scwartz et al. published the first report on transplantation of hESC-derived RPE in to a human patient in 2012 [138]. A dosage of 50,000-200,000 cells was hESC-derived RPE were administered. Initial results were promising with no indication of immune mediated graft rejection, teratoma formation or hyper proliferation at four months post-transplantation. Early data also suggested some improvements in visual acuity.

Stem cell therapy for treatment of Type I Diabetes is the newest arena for therapeutic use of hESC cells. Loss of insulin secreting cells in the pancreas is the cause of the disease. In August 2014, a diabetes clinical trial with hESC derived islet cells was approved [143]. The company Viacyte (San Diego, California) just recently reported successful surgical transplantation of their product VC-01 in to the first of 40 patients enrolled in the trial [144]. With this therapy, pancreatic precursor cells (PEC-01) encapsulated in a porous cell-impermeable membrane are placed under the skin.

Minimizing immune rejection and thus the longevity of transplanted cells is also a priority for successful use of hESC based therapies. Besides immune response from any animal derived products, there is the risk of transplantation rejection due to HLA or ABO blood group antigen mismatch. Undifferentiated hESC’s have been shown to express low levels of MHC class I molecules, which are up-regulated during differentiation [145]. The issue of donor-recipient mismatch may ultimately have to be dealt with through knockdown of MHC molecules on donor stem cells or establishment of a large number of hESC lines to allow for HLA and ABO matching [146,147]. Another intriguing possibility is the banking of ICM cells from embryos donated for research to allow for future derivation of stem cell lines with the desired match [148].

## Conclusion

Developing effective cell based regenerative therapies remain a challenge. Xeno-free culture models using extracellular matrices, biosynthetic surfaces and chemically defined media to create therapeutic grade hESC represent an important stepping stone in expanding such therapies. More data is still needed on the continued functionality of hESC cells after long term culture and continued passage in these new culture models. In depth analysis of cellular level mechanisms involved in continuous self-renewal as well as terminal differentiation will ultimately be necessary to truly harness the power of these pluripotent cells.
